# Equine PBMC Cytokines Profile after In Vitro α- and γ-EHV Infection: Efficacy of a *Parapoxvirus Ovis* Based-Immunomodulator Treatment

**DOI:** 10.3390/vaccines5030028

**Published:** 2017-09-19

**Authors:** Erika S. Hue, Eric A. Richard, Christine I. Fortier, Guillaume D. Fortier, Romain Paillot, Rudiger Raue, Stéphane L. Pronost

**Affiliations:** 1 LABÉO Frank Duncombe, Unité BioTARGen, EA 7450, Normandie Université, 14053 Caen, France; eric.richard@laboratoire-labeo.fr (E.A.R.); christine.fortier@laboratoire-labeo.fr (C.I.F.); guillaume.fortier@laboratoire-labeo.fr (G.D.F.); stephane.pronost@laboratoire-labeo.fr (S.L.P.); 2 Animal Health Trust, Centre for Preventive Medicine, Lanwades Park, Newmarket CB8 7UU, UK; romain.paillot@aht.org.uk; 3 Veterinary Medicine Research and Development, Zoetis Belgium, 1930 Zaventem, Belgium; rudiger.raue@zoetis.com

**Keywords:** Horse, Equid herpesviruses, cytokines, immunomodulator, inactivated *Parapoxvirus ovis*

## Abstract

Equine herpesviruses (EHV) infect horses early during life and the persistence of these viruses through establishment of latency represents a real risk. A better understanding of the immune response to EHV infection is necessary to improve our methods of prevention and decrease the risk of transmission. The objectives of this study were to characterise the cytokine gene expression profile of peripheral blood mononuclear cells (PBMC) after in vitro EHV-1, EHV-4, and EHV-2 infection and to determine the efficacy of inactivated *Parapoxvirus ovis* (iPPVO) against these 3 viruses. PBMC were isolated from 3 horses and infected in vitro with EHV-1, EHV-4, or EHV-2 in the presence or absence of iPPVO. In vitro culture of PBMC with EHV-1, EHV-4, and iPPVO induced a significant increase of IFN-α, IFN-β, and IFN-γ gene expression. EHV-4 also triggered a significant increase of IL-6 and TNF-α mRNA. EHV-2 triggered a significant increase of IFN-α, IFN-β, IFN-γ, IL-1β, IL-6, and TNF-α mRNA. The presence of iPPVO induced an earlier and stronger expression of IFN-α, IFN-β, and IFN-γ mRNA during EHV infection and reduced the inflammatory response induced by EHV-2. In conclusion, this study suggests that the presence of iPPVO potentiates the development of the immune response to in vitro EHV infection.

## 1. Introduction

Equine herpesviruses (EHV) are endemic viruses responsible for many clinical signs and have considerable economic impact on the horse industry. The *Herpesviridae* is divided into three subfamilies: *alphaherpesvirinae* (EHV-1, EHV-4, and EHV-3), *gammaherpesvirinae* (EHV-2, EHV-5), and *betaherpesvirinae* [1]. Among them, EHV-4 is the main α-herpesvirus responsible for acute respiratory diseases in horses. EHV-1 induces respiratory diseases as well as abortion, neonatal death, and neurological disease [2,3]. The γ-herpesvirus EHV-2 is also associated with respiratory diseases and is highly prevalent in horses [4].

**Figure 1:**
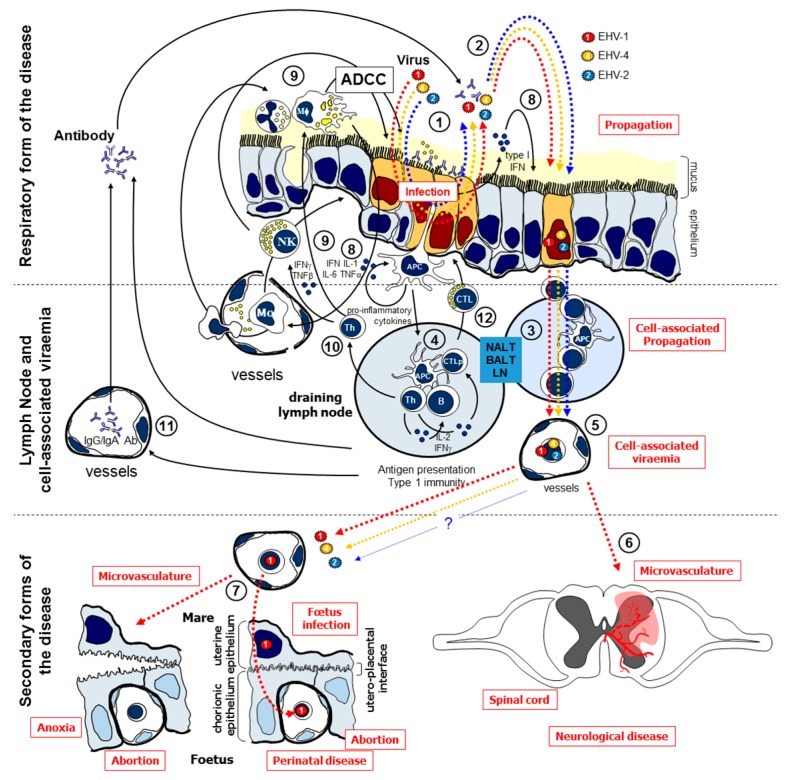
Equine herpes virus (EHV) infection and immune response, adapted from [12]. EHVs primarily infect the epithelial cells of the upper respiratory tract (1), where they replicate and are excreted (2). EHVs will reach the respiratory lymph nodes (3) where peripheral blood mononuclear cells (PBMC) will be infected (4) and the adaptive immune response will be stimulated. Circulation of infected leucocytes (5) during cell-associated viraemia disseminates EHVs to distant sites such as the central nervous system (EHV-1) (6) or the reproductive tract (EHV-1/4; potentially EHV-2). EHV-infected maternal lymphocytes reach the endometrial capillary during the cell-associated viraemia (7). EHV-1 spreads by cell-to-cell contact to the foetus through the endothelium of endometrial capillaries, the uterine epithelium, the chorionic epithelium and the endothelium of placental capillaries to finally infect foetal lymphocytes. EHV-1 can also infect endometrial endothelial cells inducing uterine pathologies, leading to a premature placenta separation and foetus anoxia. EHV-1 could also reach the central nervous system (6), infect, and damage endothelial cells, potentially leading to microvasculature and equine herpesvirus myeloencephalopathy (EHM). Infection with EHVs also induces the establishment of latency and potential reactivation of infectious virus (not represented here). In terms of immune response, the local synthesis of IFN and IL-6 by infected endothelial cells and innate immune cells will promote antiviral resistance in uninfected cells and up-regulation of major histocompatibility complex (MHC) molecules for viral antigen presentation to the immune system (8). Alveolar macrophages (Mφ) and neutrophils respond to the infection by releasing pro-inflammatory cytokines, leading to the containment of the infection, rise of body temperature, and to the recruitment of further phagocytic and natural killer (NK) cells (9). NK cells are activated by IFN-α and interleukin (IL)-12 which is synthesised by Mφ, and are cytotoxic for virus-infected cells. NK cells synthesise IFN-γ that drives the development of the adaptive immune response. The presentation of viral antigen (10) in lymphoid tissues induces the synthesis of serum (11) or mucosal antibodies able to neutralise excreted virus and to stimulate antibody dependent cell cytotoxicity (ADCC). Virus-specific cytotoxic T lymphocytes (CTL) that lyse infected cells are also stimulated (12). APC: antigen presenting cell; BALT: bronchus-associated lymphoid tissue; LN: lymph node; NALT: nasal-associated lymphoid tissue.

A characteristic of EHV is their ability to establish latency after a period of viraemia, when viruses persist subclinically in the host. In fact, latency in the trigeminal ganglia for EHV-1 and EHV-4 [5] and the lymphotropism of EHV-1, EHV-4, and EHV-2 were previously described [6,7,8,9]. Unfortunately, reactivation of latent virus, often as a result of a stress (transport, competition, immunosuppressive drugs, etc.), could be associated with clinical disease but also represents a risk of transmission and propagation to nearby animals (e.g., abortion in pregnant mare) [5,10,11]. It is therefore important to prevent the risk of reactivation and/or propagation. Several EHV vaccines have been developed but coverage and efficacy often remain insufficient [12]. EHV-1 vaccination reduces the risk of respiratory disease but shows limited or no effect against the secondary forms of diseases, such as abortion or equine herpesvirus myeloencephalopathy (EHM) [13]. No vaccine is available against EHV-2, EHV-3, and EHV-5. Currently, the therapeutic arsenal against these viral infections is limited despite significant needs in the field to prevent severe forms of these equine infections [13,14]. In this condition, the host ability to fight these pathogens is essential. At the present time, very few data are available on the immune response induced by EHV-1, EHV-4, and EHV-2 infection [15,16,17]. Generally, the secretion of type I interferon (IFN) was associated with the duration of virus shedding and could be a result of viral replication. The IFN response is an essential innate host defence mechanism against viral infection [18] as IFNs inhibit virus replication, protect surrounding cells from secondary infection, and stimulate the immune system by activating natural killer (NK) cells, macrophages, and lymphocytes (Figure 1). The use of immune modulators to boost innate immunity could help the horse to limit viraemia.

*Parapoxvirus ovis* (PPVO) is a member of the genus *Parapoxvirus* of the *Poxviridae* family. Inactivated PPVO (iPPVO) is used as an immune modulator for the prevention of respiratory disease and other infections of the horse [19]. The iPPVO was found to be effective at limiting the severity of respiratory outbreaks among horses in crowded conditions [20]. Previous studies on the horse showed immunomodulatory effects at the injection site but also in the blood after in vitro or in vivo stimulation of the peripheral blood mononuclear cells (PBMC) [21]. Preventive effects of iPPVO against herpesviruses have been reported in other species [22]. Two studies confirm these findings by describing the clinical efficacy of iPPVO on horses exposed to EHV-1 and EHV-4 by contact with carriers horses [23,24] (Studies funded by Pfizer AH/Zoetis). The effects of iPPVO on the immune response to EHV-1, EHV-4, and EHV-2 infection remain unknown in vitro.

Viraemia is a crucial step during EHV infection. A better understanding of immune modulation mechanisms taking place during viraemia is important to improve and/or develop therapeutic measures. The objectives of this preliminary study were: (1) to characterise the immunological profiles on cytokine gene expression in PBMC after in vitro EHV-1, EHV-4, and EHV-2 infection; and (2) to determine the effect of iPPVO treatment on this immune response.

## 2. Materials and Methods

### 2.1. Virus Strains and Immunomodulator

EHV-1 (Kentucky D strain, VR700), EHV-4 (405/76 stain, VR2230), and EHV-2 (LK strain, VR701) reference strains were obtained from the American Type Culture Collection. Viruses were propagated and titrated in RK13 cells by Karber method. Inactivated PPVO (iPPVO) was provided by Dr Rudiger Raue (Zoetis, Zaventem, Belgium).

### 2.2. Horses

Three horses (3 geldings, Selle français, 7–19 years old) without any clinical signs of respiratory diseases and presenting a normal blood count with absence of inflammation markers (serum amyloid A dosage) were involved in this study. No viral genome of EHV-1, EHV-4, and EHV-2 was detected in blood before PBMC collection. The study and all animal work involved have received ethical approval from the LABÉO Frank Duncombe ethical advisor (LFD-CE-06/2012) and owner consents were obtained. Blood samples were collected by an equine veterinary practitioner according to a high standard of veterinary care.

### 2.3. Culture of Equine PBMC

Heparinised blood samples were collected by jugular venepuncture. PBMC were purified by density centrifugation (Histopaque-1077, Sigma-Aldrich, Saint Quentin Fallavier, France) and washed 2 times in sterile phosphate buffered saline (PBS) (pH7.2) prior to numeration. PBMC (3 × 10^6^ cells/well) from each horse were placed in a 6-well plate and cultured in medium (RMPI 1640, Sigma-Aldrich, Saint Quentin Fallavier, France) supplemented with 5% fetal bovine serum (FBS), 200 IU/mL Penicillin and 200 ng/mL Streptomycin (Sigma-Aldrich, Saint Quentin Fallavier, France). After 20 h, before infection a low quantity of EHV-1, EHV-4, and EHV-2 viral genome were detected indicating that horses were not naïve, respectively 721 copies/mL, 2 × 10^4^ copies/mL and 1.2 × 10^3^ copies/mL. PBMC were infected with medium of EHV-non-infected cells (mock infection) or medium of infected RK13 cells with EHV-1 (multiplicity of infection (MOI) 0.01), EHV-4 (MOI 0.01), or EHV-2 (MOI 0.01), all with or without iPPVO (1:2 dilution of a solution containing 756.7 IFN units/mL). Plates were incubated at 37 °C, 5% CO_2_. Cells were centrifuged in microtubes and lysed (RNeasy minikit; Qiagen, Courtaboeuf, France) at 0 h post-infection (hpi), 18 hpi, 24 hpi, or 48 hpi. Supernatant were stored at −80 °C until further processing.

### 2.4. mRNA Isolation and Reverse Transcription

Total cellular RNA was extracted using RNeasy^®^ Mini Kit (Qiagen, Courtaboeuf, France) and treated with RNase-Free DNase Set (Qiagen) directly on RNeasy columns to eliminate genomic DNA according to the manufacturer’s instructions. RNA quality and quantity was determined using a NanoDrop^®^ 2000c Spectrophotometer (Thermoscientific, Courtaboeuf, France). Samples were stored at −80 °C until further processing. Approximately 500 ng total RNA was retrotranscribed with the Superscript^®^ First Strand System for RT-PCR (Life Technologies, Courtaboeuf, France) in combination with Oligo(dT) 12–18 primers and RNase-OUT following the manufacturer’s instructions. One sample was treated in absence of reverse transcriptase to validate the absence of genomic DNA. Three independent reverse transcriptions steps were conducted for each sample. All cDNA samples were stored at –20 °C until further processing.

### 2.5. Cytokines’ Gene Expression Analysis

Sequence-specific primers and probes used for cytokine relative quantification were previously described (Table 1). For each reaction, 2.5 µL of cDNA diluted on 1:10 was amplified in a 25 µL standard reaction (Taqman^®^ Universal PCR Master Mix, Life Technologies). The thermal cycling profile used was 10 min at 95 °C, followed by 50 cycles of 15 s at 95 °C and 1 min at 60 °C. PCRs were performed on a StepOnePlus^™^ Real-Time PCR system (Life Technologies). Data were analysed using the StepOne^™^ software v2.2.2 (Life Technologies).

All reactions were normalised to the geometric mean of two housekeeping genes (HKG), β-actin (ACTB) and glyceraldehyde-3-phosphate dehydrogenase (GAPDH). Changes in cytokine gene expression were calculated by relative quantitation using the ΔΔCt method [31] where ΔΔCt = (cytokine gene Ct − mean HKG Ct)_stimulated sample_ − (cytokine gene Ct − mean HKG Ct)_mock sample_. Treatment-induced fold changes in cytokine gene expression for each individual horse were calculated as relative quantification RQ = 2^−ΔΔCt^. Results (RQ) are expressed as the mean fold change +/− standard error of mean (SEM) in cytokine gene expression by the group of horses.

### 2.6. Quantitation of EHV by qPCR

Viral DNA was extracted from 140 µL of each culture supernatant with the QIAamp^®^ RNA viral Mini Kit (Qiagen) according to the manufacturer’s instructions.

Quantitative PCRs for EHV-1, EHV-4, and EHV-2 were developed as previously described for EHV-2 based on the standard model AFNOR NF U47-600-2 (AFNOR, La Plaine Saint-Denis, France) with the use of standard curves [30]. Each reaction was processed in a total volume of 25 µL containing 2× Taqman^®^ Universal PCR Master Mix, primers, and probes (Table 1). Thermal cycling for EHV-1 and EHV-4 proceeded at 95 °C for 10 min, followed by 50 cycles of 94 °C for 15 s and 60 °C for 1 min [28,29] and for EHV-2 proceeded at 94 °C for 10 min, followed by 45 cycles of 95 °C for 15 s and 60 °C for 1 min [30]. Quantitative PCRs were performed on a StepOnePlus^™^ Real-Time PCR system (Life Technologies). Data were analysed using the StepOne^™^ software v2.2.2 (Life Technologies).

### 2.7. Statistical Analyses

All statistical calculations were performed using NCSS9 (NCSS LLC, Kaysville, UT, USA). Normality of continuous data distribution was evaluated using the Kolmogorov–Smirnov test. The fold-changes in cytokine gene expression and viral loads were not normally distributed, as a consequence, these data were log transformed before performing a treatment comparison by repeated measures ANOVA with Tukey–Kramer multiple comparison procedure. Values of *p* < 0.05 were considered statistically significant.

## 3. Results

### 3.1. Cytokines Profiles Following PBMC Infection with EHVs

EHV-1 infection of PBMC induced an increase of IFN-γ and type I IFN mRNA expression (maximum expression at 18 and 48 hpi, respectively) and a moderate elevation of TNF-α when measured at 18 and 24 hpi. No significant effect on IL-1β, IL-6, and IL-10 mRNA expression was observed (Table 2).

EHV-4 infection increased IFN-β and IFN-γ mRNA expression by PBMC, with maximum expression when measured 48 hpi. A moderate up-regulation of TNF-α, IFN-α, IL-6, and IL-10 expression was observed after 24 h. No significant effect on IL-1β expression was observed (Table 2).

EHV-2 infection induced a large increase of IFN-γ, and IL-6 mRNA expression from 18 hpi. IL-1β, TNF-α, IFN-β, and IL-10 mRNA expression was slightly augmented. No significant effect on IFN-α gene expression was measured (Table 2).

In all these experiments, no amplification was observed in the absence of reverse transcriptase for all cytokines’ PCRs, indicating the absence of genomic DNA after extraction.

### 3.2. Cytokines Profiles Following EHV Infection and iPPVO Co-Treatment of PBMC

The treatment of PBMC with iPPVO induced a significant elevation of IFN-α (18 h post treatment, hpt), IFN-β (18–48 hpt), IFN-γ (18–24 hpt), and TNF-α (18–24 hpt) mRNA expression. A significant up-regulation of IL-10 mRNA was measured at 24 and 48 h post treatment. No significant effect on IL-1β and IL-6 mRNA expression was observed (Table 3).

**Figure 2:**
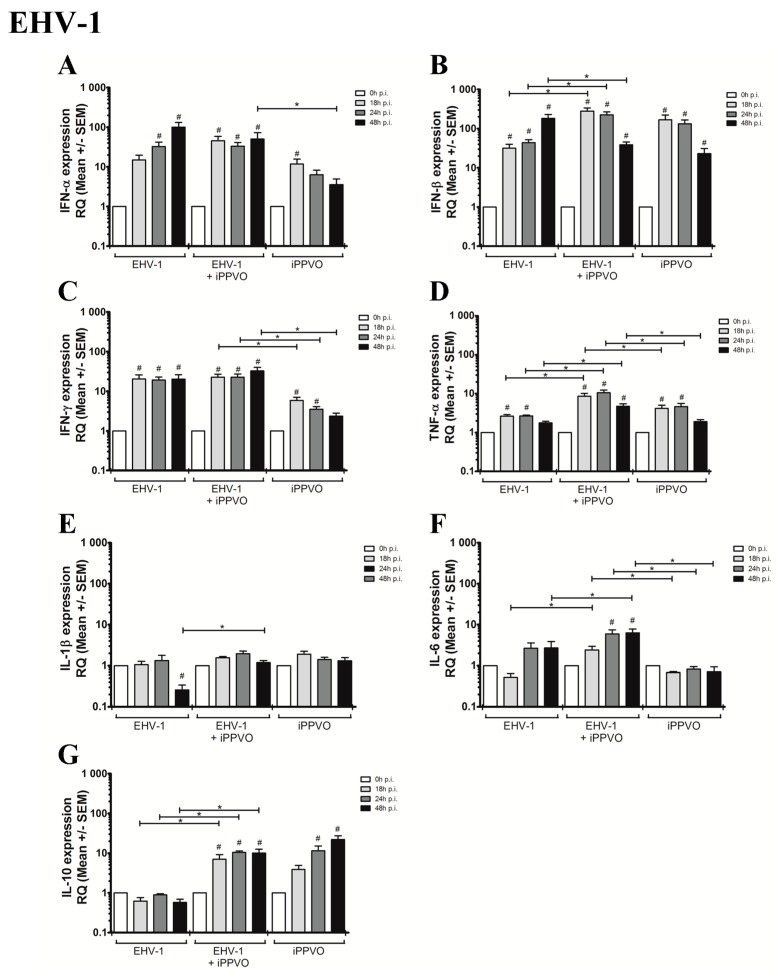
Cytokine mRNA expression measured by real-time PCR after infection of PBMC with EHV-1 alone, after co-treatment iPPVO/EHV-1, and after iPPVO treatment. Cytokines measured were IFN-α (**A**), IFN-β (**B**), IFN-γ (**C**), TNF-α (**D**), IL-1β (**E**), IL-6 (**F**), and IL-10 (**G**). White bars represent mRNA expression at 0 h (T_0_), light grey bars represented mRNA expression at 18 hpi, dark grey bars at 24 hpi, and black bars at 48 hpi; n = 3 horses with 3 independent reverse-transcriptase PCR. Data are reported as means ± SEM as fold increase over the T_0_. The level of significance was set as *p*-value < 0.05. Significant differences compared to T_0_ are indicated by # and differences between all conditions by star (*p* < 0.05). The iPPVO/EHV co-infection condition was compared with EHV infection alone or iPPVO treatment alone.

EHV-1 infection of PBMC in the presence of iPPVO resulted in a high increase of type I IFN and IFN-γ mRNA expression when measured at 18 hpi, a moderate up-regulation of TNF-α and IL-10 from 18 hpi, and IL-6 from 24 hpi (Figure 2, Table 3). No effect on IL-1β expression was observed. Compared to EHV-1 infection alone, the expression of IFN-β was significantly higher at 18 and 24 hpi (Figure 2B). The mRNA expression of TNF-α (18–48 hpi), IL-10 (18–48 hpi), and IL-6 (18 and 48 hpi) after iPPVO/EHV-1 co-treatment were also significantly higher when compared to EHV-1 infection alone (Figure 2D,G,F).

**Figure 3:**
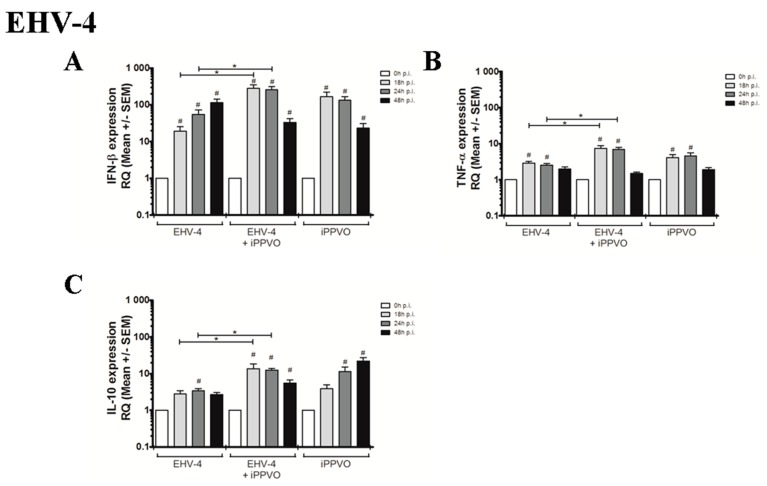
Cytokine mRNA expression measured by real-time PCR after infection of PBMC with EHV-4 alone, after co-treatment iPPVO/EHV-4 and after iPPVO treatment. Cytokines measured were IFN-β (**A**), TNF-α (**B**), and IL-10 (**C**). White bars represent mRNA expression at 0 h (T_0_), light grey bars represented mRNA expression at 18 hpi, dark grey bars at 24 hpi, and black bars at 48 hpi; n = 3 horses with 3 independent reverse-transcriptase PCR. Data are reported as means ± SEM as fold increase over the T_0_. The level of significance was set as *p*-value < 0.05. Significant differences compared to T_0_ are indicated by # and differences between all conditions by star (*p* < 0.05). The iPPVO/EHV co-infection condition was compared with EHV infection alone or iPPVO treatment alone.

Co-treatment of PBMC with EHV-4 and iPPVO resulted in an increase of type I IFN and IFN-γ (from 18 hpi) mRNA and a moderate up-regulation of TNF-α, IL-1β, IL-6, and IL-10 when measured at 18 hpi and 24 hpi (Table 3; Supplementary Figure S1). Compared to EHV-4 infection alone, IFN-β, TNF-α and IL-10 mRNA expression (measured at 18 and 24 hpi) was significantly up-regulated after co-treatment (Figure 3A–C).

**Figure 4:**
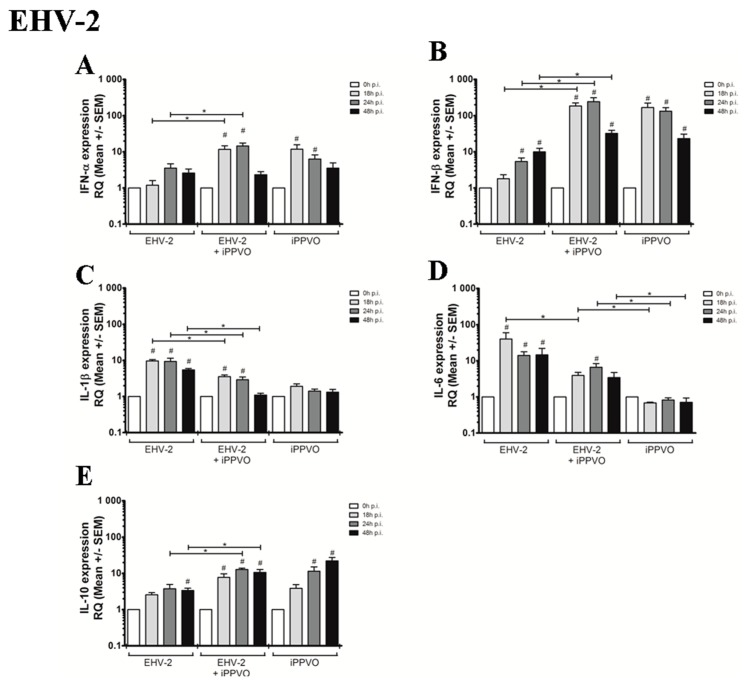
Cytokine mRNA expression measured by real-time PCR after infection of PBMC with EHV-2 alone, after co-treatment iPPVO/EHV-2, and after iPPVO treatment. Cytokines measured were IFN-α (**A**), IFN-β (**B**), IL-1β (**C**), IL-6 (**D**), and IL-10 (**E**). White bars represent mRNA expression at 0 h (T_0_), light grey bars represented mRNA expression at 18 hpi, dark grey bars at 24 hpi, and black bars at 48 hpi; n = 3 horses with 3 independent reverse-transcriptase PCR. Data are reported as means ± SEM as fold increase over the T_0_. The level of significance was set as *p*-value < 0.05. Significant differences compared to T_0_ are indicated by # and differences between all conditions by star (*p* < 0.05). The iPPVO/EHV co-infection condition was compared with EHV infection alone or iPPVO treatment alone.

The EHV-2/iPPVO combination induced a pronounced up-regulation of IFN-β and IFN-γ mRNA expression, a moderate elevation of IFN-α, TNF-α, IL-1β, and IL-10 gene expression measured from 18 hpi and IL-6 at 24 hpi (Table 3, Supplementary Figure S2). When compared to EHV-2 infection alone, the IFN-α (18–24 hpi), IFN-β (18–48 hpi), and IL-10 (24–48 hpi) mRNA expressions were significantly up-regulated (Figure 4A,B,E). Surprisingly, iPPVO treatment decreased the IL-1β and IL-6 expression in response to EHV-2 (Figure 4C,D).

### 3.3. Growth Kinetics of EHV +/− iPPVO in PBMC

**Figure 5:**
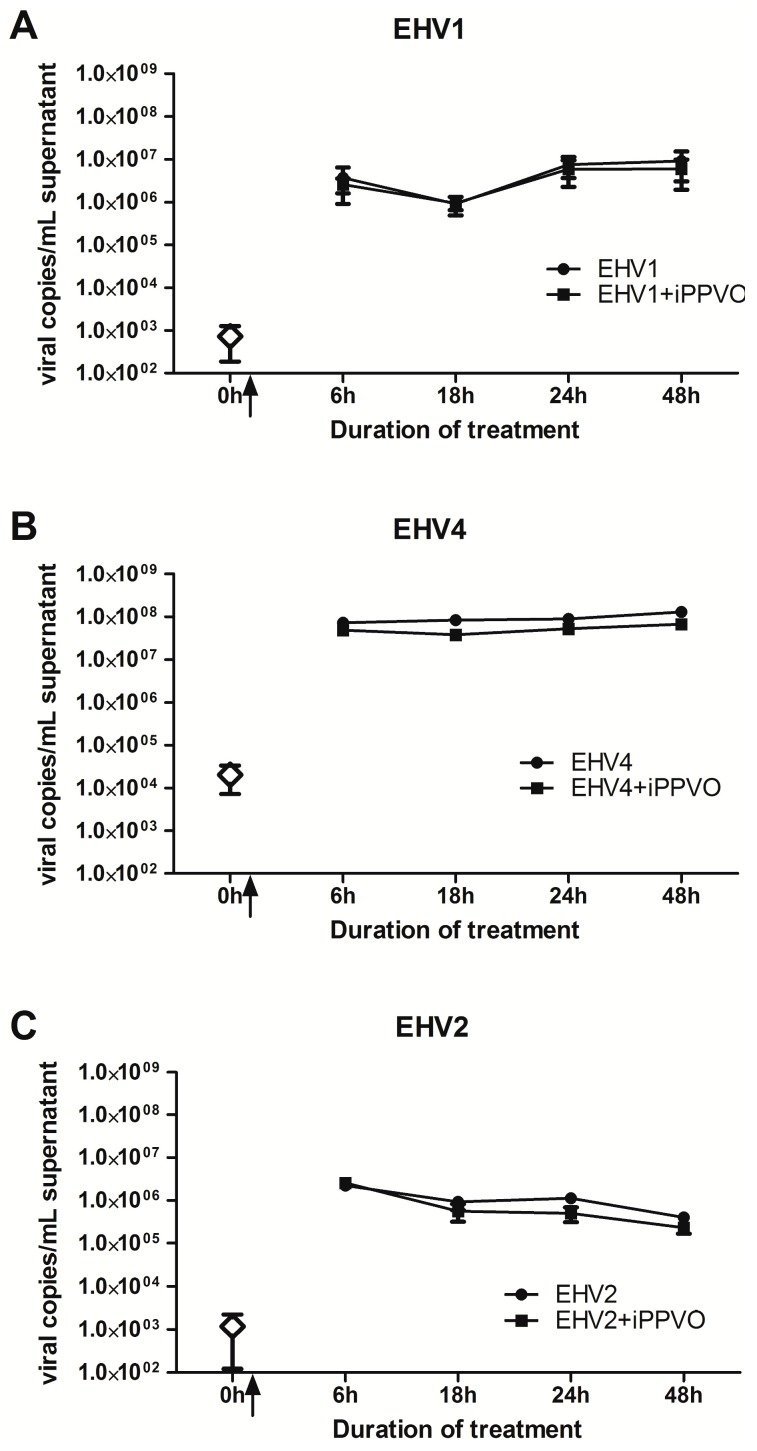
EHV titre in supernatant at different time points after (**A**) EHV-1 infection with or without iPPVO; (**B**) EHV-4 infection with or without iPPVO; **(C)** EHV-2 infection with or without iPPVO. The arrows represent the time of infection. White lozenges represent the virus genome copies detected in PBMC before infection. Data are reported as mean ± SEM viral copies/mL of supernatant.

In order to measure the effect of iPPVO on viral replication, PBMC were infected with EHV-1, EHV-4, or EHV-2 in the presence or absence of iPPVO. Viral growth kinetics were determined in culture supernatants on a 48 h period (Figure 5). No significant differences were observed between EHV-1 virus loads at 6 h compared to 48 h in the absence or in the presence of iPPVO (Figure 5A). No significant differences were observed between EHV-4 virus loads (Figure 5B) and EHV-2 virus loads (Figure 5C) at 6 h compared to 48 h in the absence or in presence of iPPVO.

## 4. Discussion

### 4.1. EHV Replication and Modulation of Cytokine Responses Following PBMC Infection

The abortive EHV-1 strain KentuckyD induced an increased mRNA expression of different cytokines such as IFN-α, IFN-β, IFN-γ, and TNF-α in PBMC after infection. This strain did not significantly modulate the mRNA expression of IL-1β, IL-6, and IL-10. These results are in agreement with the mRNA cytokines profiles previously described after infection of PBMC with the neuropathogenic EHV-1 strain Ab4 [32]. The strain Ab4 was described to increase the protein secretion of IFN-γ after PBMC infection [8,33], as also reported with the abortive strains RacL11, NY03 [34], and Army 183 [8]. Although IL-10 mRNA expression was not modulated after PBMC infection in our study or with the strain Ab4 [32], a previous study has described a moderate increase of IL-10 and IL-4 protein expression after PBMC infection with the strains Ab4, RacL11, and NY03 [34]. Moreover, EHV-1 seemed to induce different cytokine responses regarding the cell type infected. The strain Ab4 induced an increased mRNA expression of IFN-α, IFN-β, and TNF-α after infection of equine respiratory epithelial cells (EREC) [16]. IL-1β, IL-6, IL-8, and IL-12 mRNA expression were also increased in Ab4 infected EREC [16,32], whereas no modulation was described after PBMC infection [32]. The infection of equine endothelial cells with another neuropathogenic strain T953 induced an early and transitory upregulation of IFN-β mRNA expression after 12 h [35]. Differences in cytokine profiles observed with different “virus/cell type” association warrant further investigations.

PBMC infection with EHV-4 induced an increased mRNA expression of IFN-α, IFN-β, IFN-γ, and TNF-α, similar to the response observed after EHV-1 infection. EHV-4 infection also induced moderate expression of IL-6 and IL-10, contrary to EHV-1 infection. This study highlights similarity between the cytokine responses to EHV-4 and EHV-1 infection. PBMC infection with EHV-2 also increased mRNA expression of IFN-β, IFN-γ, and TNF-α, as observed after α-EHV infection. However EHV-2 induced moderate expression of IL-1β, IL-6, and IL-10. This virus has been described previously to up-regulate IFN-γ in PBMC after infection [17]. Very few data are available about the immune response induced by EHV-2.

These data are in adequacy with various immunomodulatory activities of type I IFN, which affect both innate and adaptive immune responses (e.g., increased of natural killer cell activity, promotion of type 1 T helper (Th1) response, respectively) [36]. However, as the horses are not naïve in this study, the increased IFN-γ mRNA level could be due to an adaptive immune response against EHV (T cell activity) [8,33]. IFN-γ was also described as a marker of EHV-1 specific cell-mediated immunity and cytotoxic T lymphocytes [8,33]. TNF-α, IL-1β, and IL-6 profiles correlate with an inflammatory response to EHV infection. The difference of inflammatory response between EHV-1 and EHV-4 on one hand and EHV-2 on the other hand may be related to differences between the α-EHV and γ-EHV viral replication processes or mechanisms involved in innate immunity, adaptive immunity, or reactivation. In our conditions, EHV-4 and EHV-2 induced a moderate modulation of equine IL-10. These results support evasion strategies previously described for several herpesviruses. In fact, herpesviruses can modulate IL-10 expression by expressing viral homologs of IL-10 or by activation of host IL-10, in order to regulate the inflammatory response via IL-1β and IL-6 [34,37,38]. However, no IL-10 up-regulation was observed after EHV-1 infection, which supports results previously described with the Ab4 strain [16,34]. The absence of IL-10 modulation might contribute to increased inflammation after EHV-1 infection.

### 4.2. Efficacy of iPPVO against EHV Infections in Equine PBMC

To our knowledge, this study is the first report on iPPVO-induced immune modulation investigated in in vitro in co-culture with EHV-1, EHV-4, or EHV-2. Our results tend to indicate that iPPVO does not affect the viral proliferation of EHV-1, EHV-4, and EHV-2 in vitro (measured up to 48 hpi). Looking at the increase of IFN-β expression, this result might be surprising taking into consideration recent results showing clinical efficacy of iPPVO in horses against EHV-1 infection in vivo [24]; however, the clinical effect was measured from day 11 to day 16 post-infection. The lack of iPPVO activity on EHV proliferation at 48 hpi in vitro may be linked to the shorter time of measurement compared to in vivo conditions, to the EHV-1 strain type, the viral load used in the assay, and/or inherent limitation of the in vitro model. It seems that there is a host own effect in the cytokine responses during co-treatment of iPPVO and EHV. These results are consistent with the individual/animals effects described in iPPVO treatment on PBMC from foals, human donors, and dogs [39,40,41].

Our results demonstrated that iPPVO treatment induced a high and transient mRNA expression of type I interferon (IFN-α and IFN-β) and a moderate expression of IFN-γ and TNF-α as previously described in equine PBMC [21]. The increase of IFN-γ during iPPVO treatment is likely to indicate an innate response (commercial iPPVO is not available in France, previous contact and/or administration is unlikely). Our study also showed an increased IL-10 expression but no modulation of IL-1β and IL-6 mRNA. These results confirm that iPPVO stimulates the production of cytokines in equine PBMC as it was described in other species like murine, porcine, and human [39,42,43]. iPPVO was associated with stimulation of proinflammatory, Th1, and Th2 related cytokines (reviewed by [19]). Moreover, no inhibition of the cytokine response to α-EHV and EHV-2 was observed in presence of iPPVO. The increase of IFN-α and IFN-β mRNA expression induced by EHV-1, EHV-4, and EHV-2 in the absence of iPPVO were time dependant. In the presence of iPPVO, these cytokines’ mRNA expression were advanced with maximum expression observed at 18 hpi for IFN-α and IFN-β. IFN-β mRNA expression was significantly elevated at 18 and 24 hpi in PBMC co-cultured with EHV and iPPVO compared to EHV alone. The treatment with iPPVO seemed to have no effect on the IFN-γ response after α-EHV or EHV-2 infection in vitro. However iPPVO increased the mRNA expression of TNF-α after α-EHV infection compared to α-EHV alone. These modulations were previously described with an increase of the mRNA expression of IFN-γ and TNF-α but not IFN-α and IFN-β after co-culturing with ConA [21]. Concerning inflammatory cytokines, the expression of IL-1β was not modulated in presence of iPPVO after infection with EHV-1 or EHV-4. The iPPVO significantly increased IL-6 and IL-10 mRNA expression after EHV-1 and EHV-4 infection. The expression of IL-6 was stimulated in the presence of iPPVO after EHV-1 infection but not after EHV-4 infection. The iPPVO significantly decreased IL-1β and IL-6 mRNA expression after EHV-2 infection when compared to EHV-2 alone. This result could be due to the increased IL-10 mRNA expression measured in the presence of iPPVO after EHV-2 infection.

## 5. Conclusions

The infection of PBMC with EHV-1 and EHV-4 induced rapid and strong type I and II IFN responses (IFN-α/β and IFN-γ, respectively) in vitro. EHV-4 also induced a moderate inflammatory response (TNF-α, IL-6). Two waves of cytokine mRNA expression were measured after EHV-2 infection, an early and strong IFN-γ and inflammatory cytokines response (TNF-α, IL-1β, and IL-6), followed by moderate IFN-β expression. In the presence of iPPVO, the cytokine response to EHV infection was earlier, when compared to EHV infection alone. Our study has shown a transient and moderate stimulation of innate immune and IFN-γ responses induced by iPPVO alone.

To summarise, this study suggests that the establishment of the in vitro cytokine response to EHV infection was accelerated and enhanced by the presence of iPPVO. To our knowledge, this is the first study to provide a multi-cytokine profile expressed by equine PBMC in response to EHV-1, EHV-4, or EHV-2 in vitro infection in the presence of an immunomodulator.

## Figures and Tables

**Table 1:** Primers, probes, and efficiency information: qRT-PCR of cytokines in upper part and qPCR of EHV in lower part (ACTB, actin β; GAPDH, glyceraldehyde 3-phosphate dehydrogenase; IL, interleukin; IFN, interferon; TNF, tumor necrosis factor; EHV, equid herpesvirus ; gB, glycoprotein B).

Gene	Oligo	Sequences (5′–3′)	PCR Product Size (bp)	Efficiency (%)	Sequence Accession Number	Reference
ACTB	Forward	AGCGAAATCGTGCGTGACA	70	81	NM_001081838 AF035774	[25]
	Reverse	GCCATCTCCTGCTCGAAGT				
	Probe	VIC-CAAGGAGAAGCTCTGCTATGTCGCCCT-MGB				
GAPDH	Forward	AAGTGGATATTGTCGCCATCAAT	88	90	NM_001163856 AF157626 AF035774 AF097179	[26]
	Reverse	AACTTGCCATGGGTGGAATC				
	Probe	VIC-ACTACATGGTCTACATGTTTCAGTA-MGB				
IFN-α	Forward	CCTTACTGATGGCCCTGGTG	124	87	NM_001099441; EU682378	adapted from [21]
	Reverse	ATTCTCCTCATTTGTCCCAGGA				
	Probe	FAM-CCTGCCTCACACCCATAG-MGB				
IFN-β	Forward	ACACCTGGCGTATTTTCAGAAGA	74	93	NM_001099440	[21]
	Reverse	CACAAGGAGGTTCTTAACGATGGT				
	Probe	FAM-CTAGCACTGGCTGGAATGA-MGB				
IFN-γ	Forward	AGCAGCACCAGCAAGCT	72	100	U04050; D28520; NM_001081949	[21]
	Reverse	TTTGCGCTGGACCTTCAGA				
	Probe	FAM-ATTCAGATTCCGGTAAATGA-MGB				
TNF-α	Forward	TTACCGAATGCCTTCCAGTCAAT	85	89	M64087 NM_001081819	[21]
	Reverse	GGGCTACAGGCTTGTCACTT				
	Probe	FAM-CCAGACACTCAGATCAT-MGB				
IL-1β	Forward	CCGACACCAGTGACATGATGA	64	89	NM_001082526; D42147; U92481; D42165	adapted from [27]
	Reverse	TCCTCCTCAAAGAACAGGTCATTC				
	Probe	FAM-CTTACTGCAGCGGCAAT-MGB				
IL-6	Forward	GGATGCTTCCAATCTGGGTTCAAT	65	86	NM_001082496; AF005227; U64794; EU438770	[27]
	Reverse	TCCGAAAGACCAGTGGTGATTTT				
	Probe	FAM-ATCAGGCAGGTCTCCTG-MGB				
IL-10	Forward	GACATCAAGGAGCACGTGAACTC	113	100	U38200; EU438771; NM_001082490	[26]
	Reverse	TGCTCCACTGCCTTGCTCTT				
	Probe	FAM-TGCGGCGCTGTCATCGATTTCTG-MGB				
EHV-1 gB	Forward	CATGTCAACGCACTCCCA	63	93	AY665713.1	adapted from [28]
	Reverse	GGGTCGGGCGTTTCTGT				
	Probe	FAM-CCCTACGCTGCTCC-MGB				
EHV-4 gB	Forward	GGGCTATTGGATTACAGCGAGAT	86	84	NC_001844.1	adapted from [29]
	Reverse	TAGAATCGGAGGGCGTGAAG				
	Probe	VIC-CAGCGCCGTAACCAG-MGB				
EHV-2 gB	Forward	GTGGCCAGCGGGGTGTTC	78	97	NC_001650	[30]
	Reverse	CCCCCAAAGGGATTYTTGAA				
	Probe	FAM-CCCTCTTTGGGAGCATAGTCTCGGGG-TAMRA				

**Table 2:** Cytokine (IFN-α, IFN-β, IFN-γ, TNF-α, IL-1β, IL-6, and IL-10) mRNA expression measured by real-time PCR in PBMC at 18 h, 24 h, and 48 h, after EHV-1, EHV-4, and EHV-2 infection. n = 3 horses with 3 independent reverse-transcriptase PCR. Results are presented as the means ± SEM of the expression ratio (times-fold increase over the T_0_ = 0 h). Significant differences from T_0_ are indicating in bold (*p* < 0.05).

Response	Cytokine	EHV-1				EHV-4				EHV-2			
		0 h	18 h	24 h	48 h	0 h	18 h	24 h	48 h	0 h	18 h	24 h	48 h
IFN response	IFN-α	1	14,9 (+/− 4,7)	**32,5 (+/− 9,5)**	**99,7 (+/− 31,8)**	1	5,4 (+/− 1,9)	**9,8 (+/− 2,9)**	**8,6 (+/− 3,4)**	1	1,2 (+/− 0,4)	3,5 (+/− 1,1)	2,6 (+/− 0,8)
	IFN-β	1	**31,8 (+/− 8,1)**	**43,7 (+/− 8,5)**	**182,9 (+/− 45,4)**	1	**19,1 (+/− 6,2)**	**54,8 (+/− 17,5)**	**115,7 (+/− 27,1)**	1	1,8 (+/− 0,5)	**5,4 (+/− 1,4)**	**10,0 (+/− 2,6)**
	IFN-γ	1	**20,4 (+/− 5,4)**	**19,3 (+/− 3,5)**	**20,1 (+/− 6,0)**	1	**22,0 (+/− 4,3)**	**25,2 (+/− 4,7)**	**40,3 (+/− 8,3)**	1	**25,0 (+/− 5,1)**	**25,9 (+/− 6,5)**	**18,2 (+/− 4,4)**
Inflammatory response	TNF-α	1	**2,6 (+/− 0,3)**	**2,6 (+/− 0,2)**	1,8 (+/− 0,2)	1	**2,9 (+/− 0,3)**	**2,5 (+/− 0,3)**	2,0 (+/− 0,3)	1	**3,7 (+/− 0,5)**	**3,0 (+/−0,3)**	**2,5 (+/− 0,3)**
	IL-1β	1	1,1 (+/− 0,2)	1,3 (+/− 0,5)	0,3 (+/− 0,1)	1	2,6 (+/− 0,5)	2,1 (+/− 0,6)	1,5 (+/− 0,3)	1	**9,6 (+/− 0,8)**	**9,4 (+/− 2,1)**	**5,4 (+/− 0,5)**
	IL-6	1	0,5 (+/− 0,1)	2,7 (+/− 0,9)	2,7 (+/− 1,1)	1	2,9 (+/− 0,6)	**5,7 (+/− 2,0)**	2,9 (+/− 1,3)	1	**39,9 (+/− 19,8)**	**14,1 (+/− 3,7)**	**14,7 (+/− 7,5)**
Treg response	IL-10	1	0,6 (+/− 0,1)	0,9 (+/− 0,1)	0,6 (+/− 0,1)	1	2,8 (+/− 0,6)	**3,4 (+/− 0,5)**	2,7 (+/− 0,4)	1	2,6 (+/− 0,4)	3,8 (+/− 1,2)	**3,4 (+/− 0,5)**

**Table 3:** Cytokine (IFN-α, IFN-β, IFN-γ, TNF-α, IL-1β, IL-6, and IL-10) mRNA expression measured by real-time PCR in PBMC at 18 h, 24 h, and 48 h, after iPPVO treatment and after EHV-1, EHV-4, or EHV-2 infection with iPPVO treatment. n = 3 horses with 3 independent reverse-transcriptase PCR. Results are presented as the means ± SEM of the expression ratio (times-fold increase over the T_0_ = 0 h). Significant differences from T_0_ are indicating in bold (*p* < 0.05).

Response	Cytokine	iPPVO				EHV + iPPVO											
						EHV-1 + iPPVO				EHV-4 + iPPVO				EHV-2 + iPPVO			
		0 h	18 h	24 h	48 h	0 h	18 h	24 h	48 h	0 h	18 h	24 h	48 h	0 h	18 h	24 h	48 h
IFN response	IFN-α	1	**11,8 (+/− 3,8)**	6,3 (+/− 1,9)	3,6 (+/− 1,4)	1	**45,5 (+/− 13,4)**	**33,2 (+/− 7,8)**	**50,4 (+/− 22,7)**	1	**27,4 (+/− 8,4)**	**13,5 (+/− 2,8)**	4,6 (+/− 1,2)	1	**11,7 (+/− 3,0)**	**14,5 (+/− 3,02)**	2,3 (+/− 0,5)
	IFN-β	1	**167,1 (+/− 55,4)**	**132,3 (+/− 33,7)**	**23,1 (+/− 7,8)**	1	**277,1 (+/− 56,8)**	**223,4 (+/− 45,2)**	**38,4 (+/− 7,02)**	1	**278,7 (+/− 70,6)**	**256,3 (+/− 59,4)**	**33,2 (+/− 8,5)**	1	**184,0 (+/− 40,6)**	**242,9 (+/− 69,7)**	**32,4 (+/− 6,9)**
	IFN-γ	1	**5,8 (+/− 1,2)**	**3,5 (+/− 0,6)**	2,4 (+/− 0,4)	1	**22,8 (+/− 4,2)**	**22,5 (+/− 4,6)**	**32,5 (+/− 7,6)**	1	**32,7 (+/− 7,6)**	**26,8 (+/− 3,8)**	**30,4 (+/− 7)**	1	**28,5 (+/− 4,3)**	**32,1 (+/− 7,7)**	**27,6 (+/− 6,3)**
Inflammatory response	TNF-α	1	**4,2 (+/− 0,9)**	**4,6 (+/− 1,0)**	1,9 (+/− 0,2)	1	**8,6 (+/− 1,5)**	**10,5 (+/− 1,8)**	**4,7 (+/− 0,7)**	1	**7,4 (+/− 1,4)**	**6,9 (+/− 0,9)**	1,5 (+/− 0,1)	1	**5,3 (+/− 0,7)**	**7,0 (+/− 1,6)**	1,6 (+/− 0,2)
	IL-1β	1	1,9 (+/− 0,3)	1,4 (+/− 0,2)	1,3 (+/− 0,3)	1	1,6 (+/− 0,1)	2,0 (+/− 0,3)	1,2 (+/− 0,1)	1	**3,5 (+/− 0,8)**	**2,9 (+/− 0,4)**	1,2 (+/− 0,2)	1	**3,5 (+/− 0,4)**	**2,9 (+/− 0,6)**	1,1 (+/− 0,1)
	IL-6	1	0,7 (+/− 0,04)	0,8 (+/− 0,1)	0,7 (+/− 0,2)	1	2,4 (+/− 0,5)	**6,0 (+/− 1,5)**	**6,3 (+/− 1,5)**	1	**4,1 (+/− 0,8)**	**6,1 (+/− 2,1)**	1,1 (+/− 0,3)	1	4,0 (+/− 0,8)	**6,6 (+/− 1,72)**	3,5 (+/− 1,3)
Treg response	IL-10	1	3,9 (+/− 1,0)	**11,5 (+/− 3,6)**	**22,1 (+/− 5,2)**	1	**7,02 (+/− 2,1)**	**10,5 (+/− 0,9)**	**10,0 (+/− 2,5)**	1	**13,7 (+/− 4,7)**	**12,6 (+/− 1,3)**	**5,5 (+/− 1,2)**	1	**7,7 (+/− 1,9)**	**12,7 (+/− 1,1)**	**10,6 (+/− 2,1)**

## Supplementary Materials

The following are available online at www.mdpi.com/2076-393X/5/3/28/s1.
