# Supplementary material for: Equine PBMC Cytokines Profile after In Vitro α- and γ-EHV Infection: Efficacy of a Parapoxvirus Ovis Based-Immunomodulator Treatment
Source: Vaccines (Basel). 2017 Sep 19;5(3):28. doi: 10.3390/vaccines5030028 (PMC5620559; doi:10.3390/vaccines5030028)
Supplement: Supplementary file 1 [file vaccines-05-00028-s001.pdf]

vaccines

MDPI

Article

Equine PBMC cytokines profile after *in vitro* α- and γ-EHV infection: efficacy of a Parapoxvirus ovis based-immunomodulator treatment.

Erika S. Hue [1,*], Eric A. Richard[2], Christine I. Fortier[3], Guillaume D. Fortier[4], Romain Paillot [5], Rudiger Raue[6] and Stéphane L. Pronost[7]

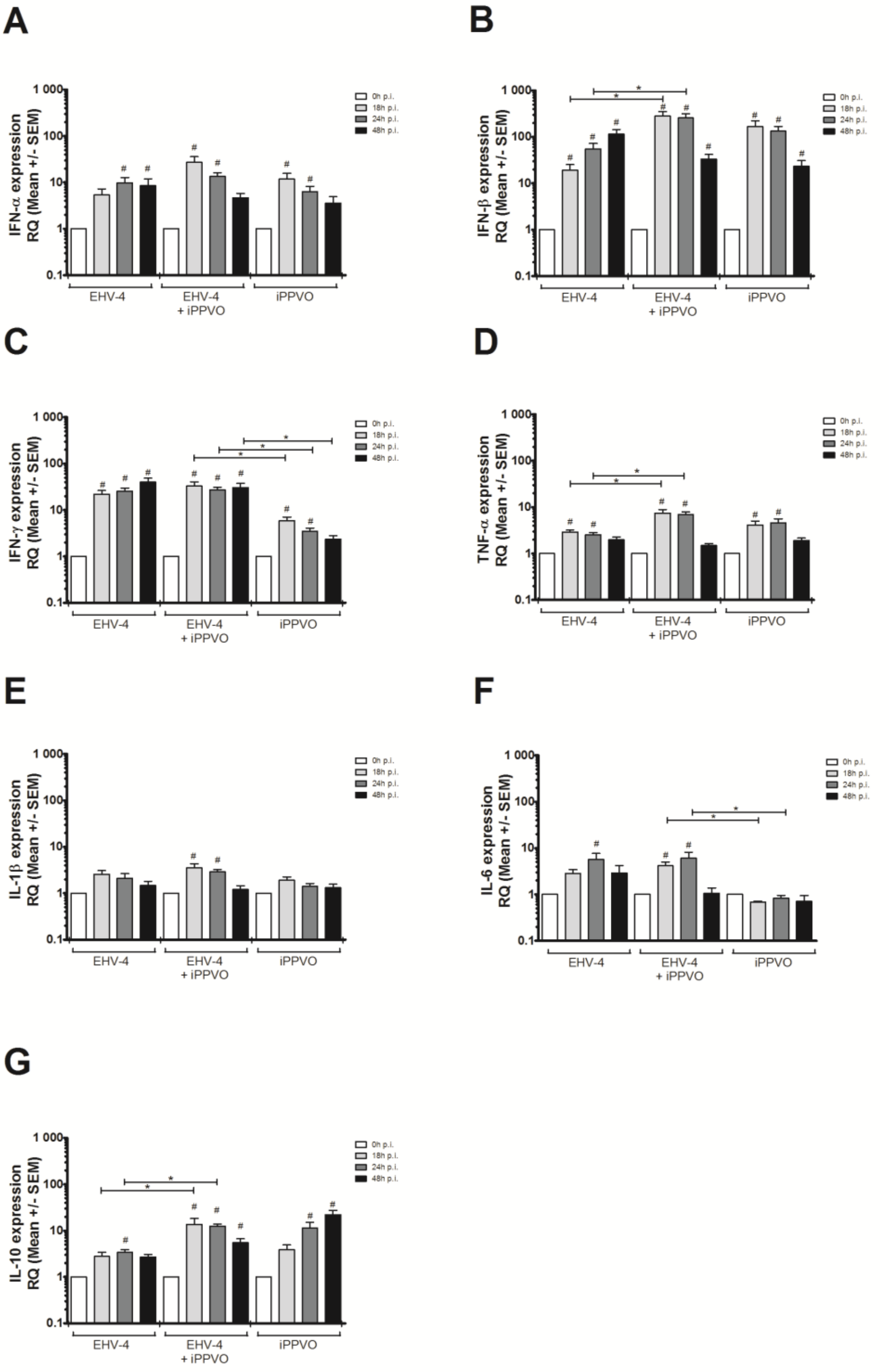

Figure S1. Cytokines mRNA expression measured by real-time PCR after infection of PBMC with EHV-4 alone, after co-treatment iPPVO/EHV-4 and after iPPVO treatment. Cytokines measured were IFN-α (A), IFN-β (B), IFN-γ (C), TNF-α (D), IL-1β (E), IL-6 (F) and IL-10 (G). White bars represent mRNA expression at 0 h (T_0), light grey bars represented mRNA expression at 18 hpi, dark grey bars at 24 hpi and black bars at 48 hpi; n=3 horses with 3 independent reverse-transcriptase PCR. Data are reported as means ± SEM as times-fold increase over the T_0. The level of significance was setup at p-value <0.05. Significant differences compared to T_0 are indicating by # and differences between all conditions by star ($p<0.05$). The iPPVO/EHV co-infection condition was compared with EHV infection alone or iPPVO treatment alone.

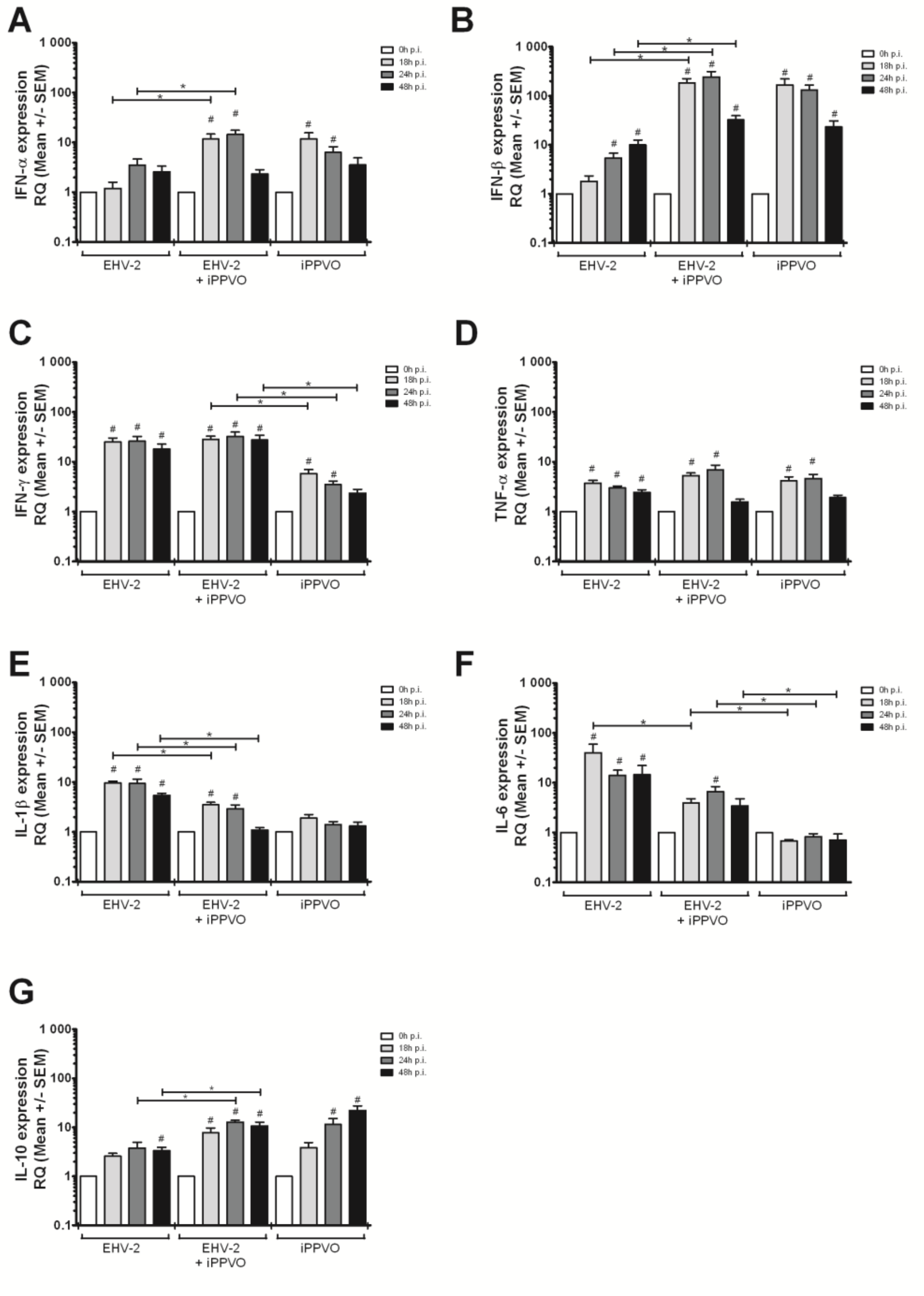

Figure S2. Cytokines mRNA expression measured by real-time PCR after infection of PBMC with EHV-2 alone, after co-treatment iPPVO/EHV-2 and after iPPVO treatment. Cytokines measured were IFN-α (A), IFN-β (B), IFN-γ (C), TNF-α (D), IL-1β (E), IL-6 (F) and IL-10 (G). White bars represent mRNA expression at 0 h (T_0), light grey bars represented mRNA expression at 18 hpi, dark grey bars at 24 hpi and black bars at 48 hpi; n=3 horses with 3 independent reverse-transcriptase PCR. Data are

reported as means ± SEM as times-fold increase over the T_0. The level of significance was setup at p-value <0.05. Significant differences compared to T_0 are indicating by # and differences between all conditions by star ($p<0.05$). The iPPVO/EHV co-infection condition was compared with EHV infection alone or iPPVO treatment alone.

© 2017 by the authors. Licensee MDPI, Basel, Switzerland. This article is an open access article distributed under the terms and conditions of the Creative Commons Attribution (CC BY) license (http://creativecommons.org/licenses/by/4.0/).
